# Carbon emissions embodied in product value chains and the role of Life Cycle Assessment in curbing them

**DOI:** 10.1038/s41598-020-62030-x

**Published:** 2020-04-10

**Authors:** Christoph J. Meinrenken, Daniel Chen, Ricardo A. Esparza, Venkat Iyer, Sally P. Paridis, Aruna Prasad, Erika Whillas

**Affiliations:** 1 Columbia University, New York, NY USA; 2 CoClear, Purchase, NY, USA; 3 University of Pennsylvania, Philadelphia, PA USA

**Keywords:** Climate change, Environmental impact

## Abstract

Life cycle-based analyses are considered crucial for designing product value chains towards lower carbon emissions. We have used data reported by companies to CDP for public disclosure to build a database of 866 product carbon footprints (PCFs), from 145 companies, 30 industries, and 28 countries. We used this database to elucidate the breakdown of embodied carbon emissions across products’ value chains, how this breakdown varies by industry, and whether the reported emission reductions vary with the granularity of the PCF. For the 866 products, on average 45% of total value chain emissions arise upstream in the supply chain, 23% during the company’s direct operations, and 32% downstream. This breakdown varies strongly by industry. Across their lifecycle, the 866 products caused average total emissions of 6 times their own weight, with large variation within and across industries. Reported achievements to reduce emissions varied depending on whether a company had reported a PCF’s breakdown to life cycle stages or only the total emissions (10.9% average reduction with breakdown versus 3.7% without). We conclude that a sector-level understanding of emissions, absent of individual PCFs, is insufficient to reliably quantify carbon emissions, and that higher reported emission reductions go hand in hand with more granular PCFs.

## Introduction

Since its debut in Europe and the US in the late 1960s, life cycle assessment (LCA)^1,2^ has been used for quantifying the environmental impacts caused throughout the life cycle of products, including industrial, commercial, and consumer products^3^. Indeed, LCA cannot only quantify a product’s current impacts, but one of the many intended purposes of LCA is to guide the (re-)design of products (including sourcing materials and the product’s production process, transportation, use, and end-of-life treatment) to render them more environmentally sustainable^4^. Of particular interest are portions of a product’s life cycle that are outside a company’s direct operations, namely the supply chain of raw materials “upstream” of the company’s own manufacturing sites and “downstream” processes such as consumer use: While these processes may be considered as being less subject to a company’s control^5^, they are often responsible for major portions of a product’s overall environmental impact and hence considered crucial levers in a company’s sustainability efforts^6^.

The quantification of a product’s life cycle carbon emissions, also referred to as product carbon footprinting^7^, is a specific form of several carbon accounting methods^8^. It can also be thought of as a limited LCA^9^ that quantifies a product’s life cycle climate change impact, but not other sustainability impacts found in wider LCA (*Discussion*)^4,7^. Here, and henceforth in this study, “carbon” refers collectively to the 6 commonly recognized greenhouse gases, which are aggregated into a single emission figure according to their relative global warming potentials (*Methods)*. Product carbon footprinting has enjoyed increasing use, due to standardized how-to guidance and improvements in the underlying methodology^5,10–13^ as well as the increased urgency to curb global carbon emissions^14^. A myriad of product carbon footprints (PCFs) for specific products have been made public – from desktop computer displays^15^ to formula milk^16^, from cars^17^ to carbon capture and storage equipment^18^, or indeed sections of a company's entire portfolio of products^19^ – and extensive LCA databases such as *Ecoinvent®*^13^, *GABI*^20^, and similar repositories have long been commercially available which store the carbon emissions (along with many other environmental impacts) of a wide range of materials (e.g., primary aluminum), energy types (e.g., electricity from photovoltaic farms), and processes (e.g., transportation by freight train). As with wider LCA, a particular interest of PCF studies has been the relative breakdown of a product’s life cycle emissions into upstream, direct operations at a company’s manufacturing site(s), and downstream. Similarly, hotspot analyses can yield remarkable insight as to where in a product’s value chain a company should focus when pursuing carbon reduction schemes. Examples for upstream hotspots are as diverse as raising cows in the life cycle of dairy milk^21^, or supplying the plastic packaging materials of bottled water products^22^. Examples for downstream hotspots include the energy consumption during the use phase of many consumer products, e.g. desktop monitors^15^. PCF can also elucidate important tradeoffs between higher upstream versus downstream emissions, as for example in the use of advanced nano materials in designing clothes^23^. In addition to these large amounts of data on individual products and materials, meta studies have analyzed types of specific products and processes, such as, e.g., the variations in relative downstream impacts of desktop computers^24^ or the impacts of utility-scale wind power^25^. In contrast, research combining many product types such as global or industry-wide studies of the effectiveness of PCFs (is there empirical evidence that carbon footprinting aids companies to lower PCFs, and by how much?) or sector-specific trends (which sectors have higher upstream or downstream emissions than others?) has been more limited. In related work – but at the organizational level rather than product level^8^ – CDP (formerly the Carbon Disclosure Project) has found that upstream emissions, averaged across all companies and industries that report this data to CDP, are twice as high as those from direct operations^26^. However, as reviewed by Blanco *et al*.^27^, said CDP data did not yet quantify all downstream scope 3 emissions. Therefore, even though companies’ upstream emissions could be compared relative to those from direct operations, it was not known how high they were as relative portions of companies’ entire emissions.

To add to above studies, here we investigate the carbon footprints of 866 products. The footprints were reported to CDP by 145 different companies, covering 30 different *Global Industry Classification Standard* (GIGS®) groups and 28 countries. The research questions we focus on are: (i) How large are typical PCFs (relative to a product’s weight) and how does this vary across industries? (ii) What portion of a products’ life cycle emissions originate upstream and downstream of a company’s direct operations, and does this breakdown of a PCF vary across industries? (iii) By how much do companies reduce their reported products’ emissions and does this correlate with the granularity and level of detail of the reported PCF?

## Results

### Overview of the product carbon footprint database

The database of product carbon footprints^7^ (PCFs) we constructed and used for this study, henceforth *PCF-Database*, is derived from carbon emissions data (measured as mass of CO_2_eq; *Methods*) for a large variety of products, from yogurt to passenger cars to soda ash. This data, available for the years 2013–2017, was collected by CDP via their Climate Change Questionnaire^28^, specifically the so called LCA portion of the questionnaire’s supply chain module (henceforth "raw data"). Note that this questionnaire refers to product “LCAs” while, more technically, the data collected specifies only on a product’s life cycle carbon emissions, not other environmental impacts. All raw data that we used in the *PCF-Database* was reported to CDP for public disclosure, with each of the 145 companies self-reporting on their product(s). With regards to data quality, 70% of footprints in the *PCF-Database* were determined by the companies using one of the three leading PCF standards (ISO^12^, GHGP^5^, or PAS2050^10^), with another 21% that did not specify the standard, and the remaining 9% determined via similar guidelines such as, e.g., the *Product Environmental Footprint (PEF)* of the European Commission^29^. While only about 35% of the reported product footprints specify whether an external audit of the footprint was carried out or not (other companies left this question blank), 67% of those were externally audited, according to the company, before being reported to CDP. Future improvements of *PCF-database* may include a more comprehensive industry and regional representation and a higher fraction of externally audited PCFs (*Discussion)*.

The raw data from CDP comprised three levels: (i) data at company level (e.g., name and *Global Industry Classification Standard* (GICS®^30^) industry classification of the company that makes the product; (ii) data at product level (e.g., the product’s name, weight (where available), and total life cycle carbon emissions as well as the reporting year); and (iii), for some products, life cycle stage data (namely total carbon emissions broken down to different life cycle stages^5^, such as raw material acquisition or use phase).

In order to enable the analyses presented in this study, we added four data elements to the raw data: (i) Product weights, thus enabling the comparison of relative carbon emissions across products of different sizes/weights; (ii) a simplified industry classification that maps 30 GICS® industry groups to 8 sectors, to allow for statistically robust sample sizes; (iii) a mapping of the large variety of life cycle stages used in the raw data to three consistent value chain portions – upstream, direct company operations, and downstream – so that origins of emissions in the value chain (hotspots) can be compared across products and sectors; and (iv) a categorization of the large variety of reasons that companies reported for changes in footprints, in order to distinguish actual changes in carbon emissions from cases in which the footprint changed merely because LCA parameters were updated (details, see *Methods)*.

The resulting *PCF-Database* contains 866 footprints. Because some footprints in the raw data were reported with more detail than others, the 866 footprints vary in granularity and in available meta data. This in essence creates several smaller datasets of the 866 footprints, each enabling different analyses. These are:

### Main dataset

A dataset that allows analyzing carbon intensity (CI) across products and 8 sectors. CI is defined as a product’s total life cycle carbon footprint [in kg CO_2_eq]^5^ per product weight [in kg] (n = 866). Note that PCFs often express emissions per functional unit (FU)^4^, e.g., a km travelled in a personal vehicle or one page printed with a color printer. However, most products in the raw data were specified only per item (e.g., one car, one printer), or possible FUs were not uniquely definable (e.g., a laptop computer). Therefore emission data per FU were not readily available. As a consequence, one product having a larger CI than another must not be interpreted as one product being more environmentally friendly (in performing the same function) than another. The 8 sectors were chosen as a balance of two objectives (*Methods)*: (i) Differentiating products of clearly different nature from each other (e.g., Chemicals versus Computer, IT & telecom); and (ii) still keeping a sufficient number of products per sector to allow for robust statistics. Therefore, sectors were defined based on GICS industry groups, but some sectors combined multiple such industry groups together whose underlying products comprise various types (e.g., Home durables, textiles, and equipment).

### Subset A

A subset of the 866 footprints, which allows analyzing CI as well as its breakdown into the three value chain portions (upstream, direct company operations, downstream) across sectors (n = 421). Some of the 421 footprints further specify the emission contribution associated specifically with upstream and downstream transportation (n = 298) and/or the contribution associated specifically with end-of-life (n = 180). However, for footprints whose transportation and/or end-of-life contribution are not specified, these emissions are not zero, but already included in the value chain portions (namely upstream and/or downstream for transportation, and downstream for end-of-life).

### Subset B

Another subset of the 866 footprints, which allows analyzing the company’s self-reported change in PCF vis-à-vis its previous assessment, typically 1–2 years prior (n = 250).

Table 1 shows an overview of the 3 datasets along with their respective sample size (i.e., number of footprints), broken down by sector. All statistical analyses and statistical robustness measures such as standard error of the mean (SEM) considered the exact size of each respective subset (*Methods*).

**Table 1:** Sample sizes of the three datasets in PCF-Database, organized by available granularity of data. [] Show number of cradle-to-gate footprints, i.e., whose downstream emissions were not assessed by the company.

Detail included with footprint	Main: CI	Subset A: CI & value chain breakdown			Subset B: CI & footprint change	
	Product weight and CO_2_ eq	Upstream, direct operations, downstream	Contribution from transport	Contribution from end-of-life	Footprint change	Footprint change and reason for change
Automobiles & components	75	12 [0]	4	10	7	5
Chemicals	116	39 [28]	14	0	42	30
Commercial equipm. & capital goods	56	35 [0]	31	20	19	19
Computer, IT & telecom	253	161 [8]	141	104	54	44
Construction & commercial materials	67	44 [17]	27	0	45	41
Food & beverage	139	70 [3]	67	14	54	50
Home durables, textiles & equipm.	122	35 [1]	12	32	23	17
Packaging for consumer goods	38	25 [23]	2	0	6	6
**All sectors**	**866**	**421 [80]**	**298**	**180**	**250**	**212**

Such footprints create a bias towards smaller CI and were therefore excluded from some analyses (*Methods*). All 421 footprints with value chain breakdowns include the 3 emissions portions from upstream, direct operations, and downstream processes. Some of these footprints further split out emissions specifically for transport (which are also included in up- and/or downstream) and/or end-of-life (which are also included in downstream).

### Ranges of product footprints, weights, and CIs

For the 866 footprints in the *PCF-Database*, product weights range from 1.3 grams to 600 metric tons. Total embedded carbon emissions range from 0.4 g CO_2_eq to 3,718 metric tons CO_2_eq. CI varies from 0.11 to 973, i.e. by nearly 4 orders of magnitude. The distribution of CIs is highly asymmetric (median 5.4, arithmetic mean 34, skew 5.6, kurtosis 41) and is well approximated by a lognormal distribution of μ = 1.85 and σ = 1.95 (*Supplementary Information*). Therefore, all subsequent statistical analyses of CIs such as means, standard error of the means (SEM), t-tests, ANOVA, and regressions are carried out on ln(CI).

### CI by industry sector

**Figure 1:**
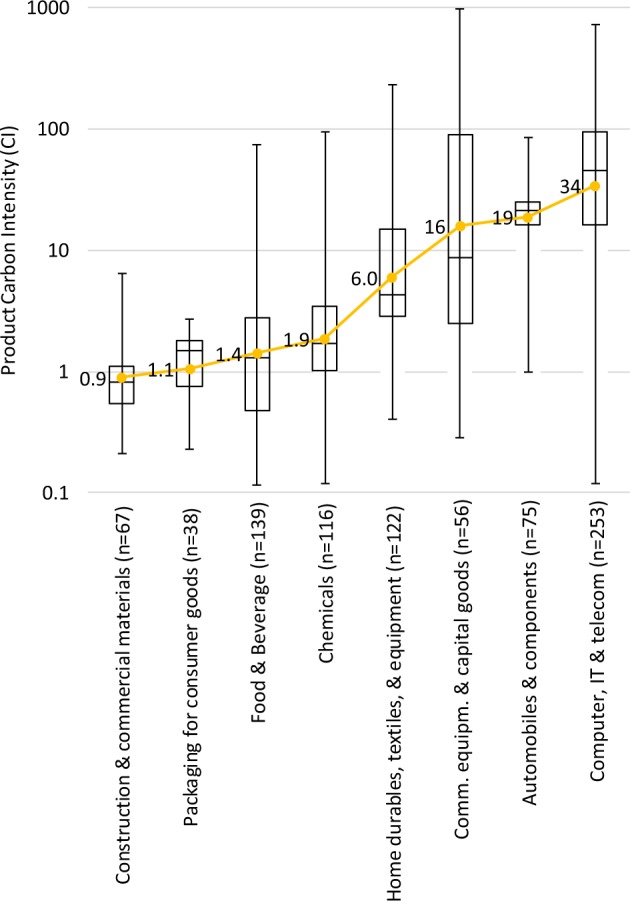
CIs broken down by sector. For each sector separately, the CI distribution is illustrated via box-plots, showing minimum CI, 25^th^, 50^th^ and 75^th^ percentiles, and maximum CI of each sector. Numbers in parentheses at the end of each sector name indicate the sample size as per Table 1. Yellow line and numerical labels show the (geometric) average CI per sample. SEMs of average CIs (*Methods*) are small, between 7–13% for all sectors, except for the sector *Commercial equipment and capital goods* which has an SEM of 29% (SEMs not plotted).

Given the large range of CIs, next we sought to understand how much average product CIs vary by sector. The range of average CI *between* sectors is large, from 0.9 to 34 (Fig. 1). These differences in sector averages are statistically significant for most sectors, with pairwise student t-tests (two-tailed) yielding p-values < 0.05 for 24 of the 28 pairwise sector comparisons. Only 4 such comparisons do not show statistical significance (namely sectors with mean CI 0.9 vs. 1.1, 1.1 vs. 1.4, 1.4 vs. 1.9, and 16 vs. 19). Despite these sector-wide trends, the range of smallest to largest CIs *within* a sector is also large, typically 1–3 orders of magnitude. Accordingly, *inter*-sector variance of CI is only slightly larger than *intra*-sector variance (ANOVA, η^2^ = 0.53).

### Breakdown of carbon footprints across value chain – all footprints

On average, the majority of products’ carbon emissions arise upstream (44.5% ± 1.5% of total emissions), followed by downstream (32.3% ± 1.6%) and only the remainder (~23%) from direct operations (n = 421). When excluding cradle-to-gate footprints from this analysis (which by definition have no downstream emissions and therefore higher relative upstream and direct contributions), these portions change to: Upstream 40.0% ± 1.6%, downstream 39.9% ± 1.8%, and direct operations ~20% (n = 341). Based on the subsample of footprints that were reported with separate data on emissions from transport (part of upstream or downstream) and end-of-life (part of downstream), average transport emissions are 7.6% ± 0.7% (n = 298) of the total footprint, and average end-of-life emissions are 3.9% ± 0.6% (n = 180). In summary, on average less than 1/4 of a product’s total carbon emissions arise during a company’s direct operations. This underlines that the often recognized, crucial role of up and downstream processes in understanding overall carbon emissions holds across a wide variety of products and sectors (*Discussion*).

### Breakdown of carbon footprints across value chain – by sector

**Figure 2:**
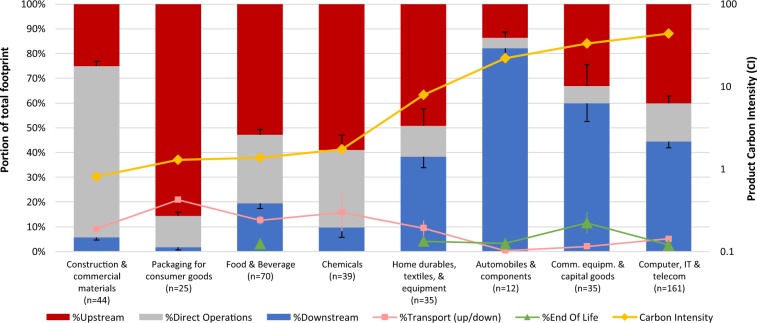
Emissions breakdowns along a product’s value chain, by sector. Sectors are sorted, left to right, by increasing average CI. Numbers in parentheses at the end of sector labels indicate sample size as per Table 1. Error bars show SEMs (*Methods*), with only one side of the symmetric error bars shown, to avoid cluttering the graph. For 3 sectors, the end-of-life portion could not be separately quantified because those sectors did not include any products with the respective breakdown for end-of-life emissions.

As shown in Fig. 2, value chain breakdowns vary strongly by sector. For example, products in *Packaging for consumer goods* incur the highest average emissions from upstream processes (85.6% ± 1.1%, n = 25), *Construction and commercial materials* from direct operations (69.5% ± 1.5%, n = 44), and *Automobiles & components* from downstream processes (82.2% ± 3.4%, n = 12). However, *intra*-sector variance of hotspots is even larger than the *inter*-sector variance, as evidenced by η^2^ = 0.23 (ANOVA) for the portion of upstream emissions, η^2^ = 0.45 for the portion from direct operations, and η^2^ = 0.35 from downstream emissions (n = 421). This shows that, while sector trends do exist, a mere sector-level understanding of product carbon hotspots is insufficient to understand the value chain emission breakdown of individual products within sectors (*Discussion*).

### Relationship between value chain hotspots and total CI

Comparing a sector’s average downstream emissions to its average CI in Fig. 2. shows a pattern: Sectors with carbon hotspots in downstream processes (i.e., with a large average downstream portion of PCFs) tend to have higher average CI. To investigate this pattern in more detail, and at the level of individual products rather than sectors, we analyzed the correlation between ln(CI) of a product on one hand and the various value chain portions of total emissions on the other hand. To avoid a bias, in this analysis specifically, we excluded cradle-to-gate footprints (*Methods*) whose downstream emissions are not counted altogether and which are therefore expected to have lower CI than other otherwise comparable products whose downstream emissions are counted.

**Figure 3:**
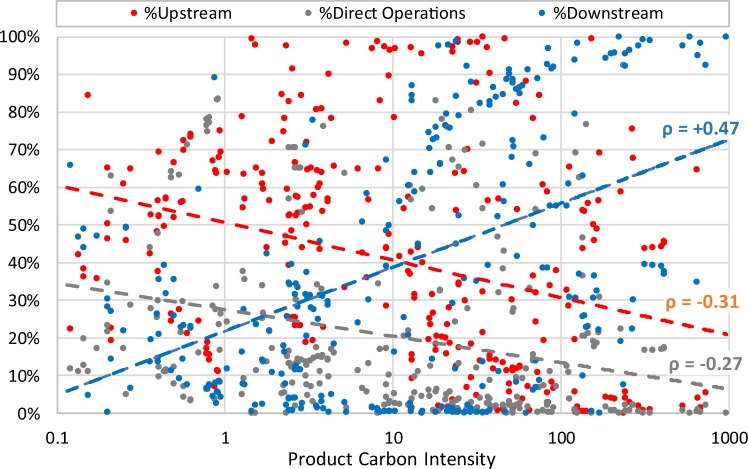
Emission breakdowns vs. CI. Each of the 341 product footprints are represented as three dots, indicating the product’s CI (x-axis) versus the 3 portions of upstream, direct operations, and downstream emissions (y-axis). Dashed lines show the three respective linear regressions between ln(CI) and the portion of total emissions (using the same color code). Excluded from this analysis are cradle-to-gate footprints (*Methods*) whose downstream emissions are not counted (i.e., 0% of total) and which are therefore expected to have systematically lower CI.

As can be seen in Fig. 3, ln(CI) has a statistically significant positive correlation with the portion of downstream emissions (ρ = +0.47, n = 341, p < 0.05), whereas this correlation is negative for the portion of upstream emissions (ρ = −0.31, p < 0.05) and direct-operation emissions (ρ = −0.27, p < 0.05). In other words, in addition to other factors affecting CI, products with higher CI exhibit (on average) a statistically significant shift in carbon hotspots from upstream processes and direct operations to downstream processes. While products with higher CI might still have higher emissions per product weight everywhere in their entire value chain, the increasing *portion* of downstream emissions to total emissions implies that larger CIs tend to go hand in hand in particular by higher emissions from downstream processes. This gives downstream processes a particular role in understanding individual, large product CIs, even though downstream emissions, on average, contribute the same emissions as upstream processes (both ~40% in this sample). While no causation can be proven via this relationship, it has important ramifications for efforts to curb emissions through changing a product’s downstream processes (*Discussion*).

In order to test for the possibility that the above product-level correlation between a product’s CI and its downstream emission hotspot is simply a reflection of the sector pattern already seen in Fig. 2 (as opposed to a pattern for individual products), we also analyzed these correlations within each sector separately. The footprints in 5 of the 8 sectors show a positive correlation between ln(CI) and the downstream emission portion, ranging from ρ = 0.16 to ρ = 0.94 (however, only 3 of these are statistically significant at p < 0.05 because of the smaller sample sizes when analyzing sectors separately). 2 of the 8 sectors, *Construction & commercial materials* and *Food & beverage*, have statistically significant *negative* correlations between ln(CI) and the portion of downstream emissions. In these 2 sectors, the positive correlation with ln(CI) is instead with the portion of direct operation emissions. For *Construction & commercial materials* the correlation between ln(CI) and the portion of direct operation emissions is ρ = 0.40 (p < 0.05) and for *Food & beverage* it is ρ = 0.69 (p < 0.05). The last sector, *Packaging for consumer goods* did not allow for this correlation analysis because it had only 2 footprints that were not cradle-to-gate footprints. In summary, for most sectors, higher than average CIs within sectors are associated with higher than average emissions in downstream processes. In contrast, for *Construction & commercial materials* and *Food & beverage*, higher CIs are associated with higher emissions from direct company operations (*Discussion*).

### Carbon reduction achievements

250 product footprints were reported along with a relative increase or decrease in footprint, as compared to the company’s previous internal assessment of the product (typically 1–2 years prior*)*. We categorized these according to the reported reason for the footprint change (see *Methods* for specific product examples and their reported reduction achievements).

84 of the 250 reported changes were due partially or wholly to updates in LCA methodology and/or model parameters (rather than reported changes in the product’s life cycle emissions) or were reported without a specified reason altogether. The range of reported changes in this category is large (from a footprint reduction of 82% to an increase of 57%). However, the average reported footprint change in this category is statistically indistinguishable from zero (−0.9% ± 2.3%, n = 84). This is consistent with the fact that the majority of these changes were due simply to updates in the LCA model – which should have no upward or downward trend – but not to reported changes in emissions anywhere in the product’s value chain.

The remaining 166 footprint changes range from −81% (emission reduction) to +103% (emission increase). The average change was −7.5% ± 1.7%. This shows that, on average, companies reported substantial reductions in life cycle carbon emissions within a 1–2 year time frame, reflecting the various initiatives reported in the raw data (e.g., using less carbon intensive raw materials, more efficient manufacturing, or reducing a product’s electricity consumption during its use phase). However, it is difficult to determine to what extent the sample of 166 products is representative of all 866 footprints. If there was a reporting bias – such as that companies more likely reported a change when it showed a substantial emission reduction, but otherwise left the respective section of the questionnaire blank – then the 7.5% average reduction would overestimate the reductions that companies determined in the larger sample.

**Figure 4:**
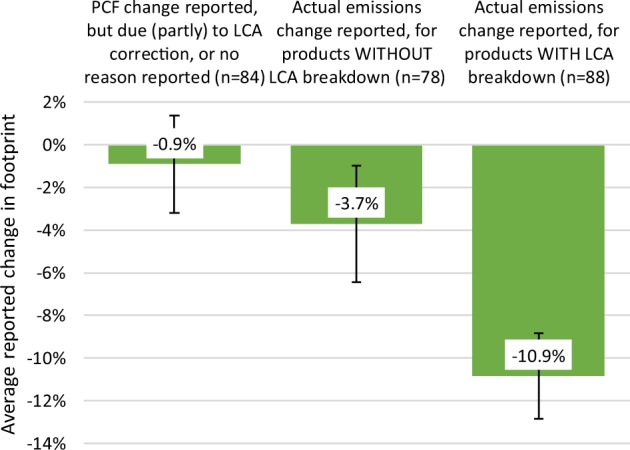
Average reported carbon reduction achievements by category. The 250 reported footprint changes were sorted into 3 categories, reflecting different underlying reasons for the reported changes as well as different granularity of the reported product footprints. Error bars show ±1 SEM around the arithmetic mean. The difference in average achieved reductions in the two samples (footprints *with* versus *without* life cycle stage information) is statistically significant (two-tailed student t-test, unequal variances; p < 0.05).

In an effort to control for such a bias, next we sought to understand whether those companies that reported not only a product’s total footprint, but in addition its breakdown to life cycle stages reported on average steeper reductions than other companies that reported a product’s total footprint only. The underlying hypothesis was that companies with a better and more granular understanding of their product’s life cycle were able to leverage the insights afforded by LCA to steer their various emissions reduction initiatives towards those that yield the highest reductions along a product’s value chain (*Discussion*). We found that the average reported emission change for footprints that included life cycle stage breakdowns was −10.9% ± 2.0% (n = 88), about twice the reduction as for footprints that were reported without valid stage breakdowns (−3.7% ± 2.7%; n = 78). The results are summarized in Fig. 4.

### Average CI

The (geometric) average of the 866 CIs is 6.3 with an SEM of ±7%. In other words, across its lifecycle, an average product causes total embedded carbon emissions of 6.3 times its own weight. In order to check for possible biases in this figure from sectors with higher or lower product representation amongst the 866 products (Table 1), we also calculated the geometric average CI directly from the 8 sector averages (rather than the number-weighted average CI of all 866 products). This calculation yielded a CI of 5.3. The possible limited representation of products in the database of the wider economy – both sectorally and regionally – is addressed in *Discussion*.

## Discussion

### Conclusions and relevance for companies’ PCF reduction strategies

We presented a side by side comparison of 866 life cycle product carbon emission intensities (CI) across a wide range of industry sectors, from 145 companies in 28 countries. Average CI across all 866 products was 6.3, meaning that, on average, a product causes life cycle carbon emissions of 6.3 times its own weight. While CI varies strongly (and statistically significantly) across sectors, about half of the variance in CI occurs within sectors, indicating that a sector-level understanding of a product is not sufficient to quantify its embedded carbon emissions.

Our results show that a detailed understanding of a product’s life cycle, particularly when including a granular breakdown of processes up stream and downstream of a company’s own operations, not only aids in predicting the product’s associated carbon emissions, but is also correlated with steeper reported reduction achievements. The results thus add empirical evidence to what proponents of sustainable product supply chains^6^ and LCA approaches have argued for many years^4,5,31^. The average reported reduction in product carbon emissions (7.5% ± 1.7%) is substantial, but this average could be affected by a reporting bias (*Results*). However, such possible bias in overall reductions notwithstanding, companies that reported product footprints along with their breakdown to life cycle stages reported reduction achievement about three times higher than those that reported footprints without such granular breakdown (10.9% vs. 3.7%). There are several possible reasons why some footprints may have been reported without breakdown – the company did not know the product’s more detailed footprint, was not confident in it, or simply did not have sufficiently knowledgeable staff on hand when reporting the footprint to CDP. However, we argue that all these reasons are consistent with the following, possible interpretation: Companies that overall had a better understanding of their product’s carbon emissions along its life cycle achieved larger reported carbon reductions.

Our second set of results relates to where in a product’s value chain most carbon emissions arise. Our results, which were determined at the *product-level*, are consistent with a study by CDP^26^, which found that in annual, *company-level* carbon emission data, upstream emissions are twice as high as those from direct operations (average across industries). However, said CDP analysis did not yet quantify all downstream emissions, so the actual portions of companies’ total emissions could not be determined and can therefore not be compared to our study. In our study, emissions related to downstream processes comprised 32% of total value chain emissions (40% when excluding cradle-to-gate footprints; *Methods*).

While downstream emissions, on average, are lower or equal to upstream emissions, it is downstream emissions in particular that appear to be associated with higher than average CI in 6 of the 8 assessed sectors (exceptions: *Construction & commercial materials* and *Food & beverage*). This could point to a particularly large potential for companies to curb product carbon emissions via initiatives that target downstream processes (e.g., lower energy consumption in the use phase, lower waste impacts), even for those products and sectors whose downstream processes do not contribute the largest portion of total emissions.

### Limitations and future work

Owing to the makeup of CDP’s member companies, the product set in the current *PCF-Database*, while quite large (866), is dominated by companies headquartered in USA and Canada (311 products), Europe (259), and Japan (110). In contrast, only 6 products are from companies headquartered in the world’s 2^nd^ largest economy, China. This shows that *PCF-Database*, despite 866 total products, still lacks in regional representation. We expect that as CDP’s member base as well as the participation particularly in CDP’s Climate Change Questionnaire^28^ Supply Chain Module increases, the regional mix of products will be more akin to respective economic activity. Similarly, of the 30 GICS® industry groups in *PCF-Database*, only 3 groups make up about half of all footprints in *PCF-database* – 195 in Technology Hardware and Equipment, 161 in Materials, and 101 in Food, Beverage & Tobacco – indicating room to improve industry representation.

This raises the question whether our results are skewed towards sectors that happen to have more footprints in *PCF-Database* (e.g., ~30% of the 866 footprints are in Computer, IT, and telecom; Table 1). To check this, we also calculated the average CI and its value chain breakdown as an average of the 8 sector averages directly (rather than the number-weighted average across all footprints). We found a (geometric) average of 5.3 for CI and an (arithmetic) average value chain breakdown of 45% upstream, 22% direct operations, and 33% downstream emissions. The close agreement with the number-weighted averages presented in *Results* show that the uneven distribution of footprints across sectors did no materially skew our results.

Finally, the reliability of individual, self-reported PCFs – irrespective of the company’s regional origin or sector – will further improve if and when more companies request external audits of their PCFs before submitting them to CDP.

With respect to product sustainability generally, as valuable and increasingly popular PCFs may be, they address only one aspect of a product’s wider environmental and social impacts (namely climate change). LCA, in contrast, typically assesses a wider range of sustainability aspects. This lowers the risk of so called problem shifting^7^, in other words the risk that a company may succeed in reducing a product’s carbon emissions, but renders the product’s life cycle worse in other aspects. Including other product sustainability aspects beyond carbon, as propagated by Product Environmental Footprints^29^, may therefore be considered going forward.

## Methods

### Overview: From CDP’s raw data to the *PCF-Database* developed and used in this study

*PCF-Database* is derived from data on greenhouse gases (GHG) caused throughout the life cycle of products. GHG are quantified as CO_2_eq, a measure of equivalent global warming potential across the 6 commonly recognized GHG^5^, and are henceforth referred to simply as “carbon emissions”. Companies reported these emission data, available for years 2013–2017, via CDP’s Climate Change Questionnaire^28^, specifically the product life cycle assessment (LCA) portion of the questionnaire’s Supply Chain Module (henceforth “raw data”). Any raw data used in this study was reported to CDP for public disclosure.

To enable the specific analytics presented here, we carried out the following four steps which added four data elements to the raw data. Rationale and a brief summary for each step is given in this overview. Details on each step are provided in the subsequent sections. *PCF-Database* and sample sizes are summarized in Table 1 (*Results*).

*Step 1:* Where not already included in the raw data, the weight of each product was determined through third party sources. This allowed us to define a carbon intensity for each product (kg CO_2_eq (self-reported by company) per kg of product, henceforth CI) to enable emission comparisons across products of different weight/size. This yielded a sample of 866 product CIs.

*Step 2:* The 30 *Global Industry Classification Standard* (GICS®^30^) industry groups in the 866 CIs were mapped to a less granular set of 8 sectors, striking a balance between similarity of products within sectors on one hand and statistically robust sample sizes per sector on the other (GICS mapping available in *Supplementary Information*).

*Step 3:* The large variety of life cycle stage taxonomies used in the raw data were mapped into one of three portions of a product’s value chain: (1) upstream (i.e. supply chain, e.g. acquisition and preprocessing of raw materials); (2) the reporting company’s own direct operations (e.g., factory energy consumption while assembling the product); or (3) downstream (e.g., energy consumption during the product’s use)^28^. In addition, where possible, life cycle stages were also identified as exclusively transport (yes/no) or exclusively end-of-life (yes/no). This allowed us to break down 421 of the 866 CIs into their relative portions of emissions from upstream, direct operations, and downstream processes (henceforth, value chain breakdown). For the other 445 of the 866 footprints, the reporting company had provided no or in-valid detail on life cycle stages, thus precluding such breakdown (stage mapping available in *Supplementary Information*).

*Step 4:* The detailed reason a company reported as having caused a recent change in the footprint – vis-à-vis their previous assessment, typically 1–2 years prior – was mapped into 4 categories.

The remaining footprint submissions to CDP’s questionnaire were for services^5^ (e.g., carbon emissions per one hotel guest per night) or incomplete (e.g., product name missing or incomplete and therefore weight undeterminable; carbon emissions left blank). Services were excluded as outside the scope of the current study (no weight can be associated and therefore CI is unavailable which in turn precludes meaningful comparisons across footprints). Therefore, only said 866 footprints were included in the *PCF-Database* and analyzed in this study.

Note that above sample sizes count a single product whose footprint was reported in more than one year as multiple footprints. This was done with the following rationale: First, the majority of products are anyway reported in only one of the 5 years. The sample of 866 footprints thus comprises 614 unique product names. Second, counting products rather than footprints would have required distinguishing cases where a product underwent a complete change from one year to the next, essentially creating a different product (without however changing its name) from the opposite cases where the same product (including the same up- and downstream life cycle) was reported under a modified or even completely new name. The CDP raw data does not include sufficient detail to allow such distinctions (such as non-ambiguous product identifications based on numbers/codes).

### Step 1: CIs and associated sample of 866 products

In 2013–2017, companies from 50 countries covering all 5 continents submitted a total of 1,597 footprints to CDP. Of these 1,597, 194 were blank submissions (without product name and/or emissions data), 263 were for services (excluded, see above), and 197 were incomplete submissions (emissions specified but product detail insufficient to determine weight of product; e.g., “office printer” without model number or other indication of size or weight). Of the 943 remaining submissions, the raw data included the weight for 361 products (e.g., footprint for 1 metric ton of soda ash or footprint for one 24” monitor with a company-specified mass of 7.4 kg). For the remaining 582 footprints, we determined the (gross) product weight via third party sources such as manufacturer brochures, specification sheets, or weights provided by online retailers. For about half of the 582 footprints, product weights were available from more than one such source, and these weights agreed to within ±5–10%. This uncertainty is much smaller than the ranges of CI in each sector (see Fig. 1) and was therefore treated as negligible in subsequent analyses. Of the 943 resulting CIs, 44 were below 0.1, and 33 were above 1,000, i.e., well outside the range of typical CIs, both in *PCF-Database* (*Supplementary Information*) or other databases such as *Ecoinvent*^13^. These 77 were excluded based on the rationale that they are probably simply incorrect (e.g., a footprint was reported in grams or tons instead of kg), or, in rare cases, are correct, but would constitute outliers in the subsequent statistical analyses. For example, the complex manufacturing process to make one milligram of a pharmaceutical drug may cause embedded carbon emissions of more than 3 orders of magnitude of its weight, but the resulting CI of above 1,000 is atypical of any other chemicals in the CDP data.

### Step 2: Mapping to 8 industry sectors

For 2013–2015, each submitting company in the CDP raw data had an associated GICS® industry group and GICS® industry. In the CDP questionnaire for 2016 and 2017, these were replaced with the Global Reporting Initiative’s (GRI) Business Activity Groups. We first mapped the GRI classifications in 2016–17 back to GICS®, following GRI’s mapping^30^. We then created a new mapping (included in *Supplementary Information*) from the combination of GICS® industry group and GICS® industry to a set of 8 sectors defined for the *PCF-Database*: Construction & commercial materials; Packaging for consumer goods; Food & Beverage; Chemicals; Home durables, textiles, & equipment; Automobiles & components; Comm. equipment & capital goods; Computer, and IT & telecom. These 8 sectors were chosen according to the following criteria: (i) Providing reasonable granularity with respect to sectors (e.g., chemicals differentiated from computers); but still (ii) allowing large enough sample sizes per sector (ranging from 38 to 253 footprints per sector) to reveal statistically significant sector trends (see *Results*). For most companies, the mapping was straight forward based on simply the GICS® codes (e.g., GICS® Food, Beverage & Tobacco|Beverages was mapped to Food & Beverage). In some cases, the mapping was further informed by the nature of the reported products (e.g., Materials| Containers & Packaging was mapped to Packaging for consumer goods (typical products in the raw data were PET bottles) whereas Materials|Paper and Forest Products was mapped to Construction and Commercial Materials (typical products in the raw data were intermediate paper or cardboard production, but not finished packaging).

### Step 3: Breakdown of CI into value chain stages

Of the 866 footprints, 454 (52%) included separately reported carbon emissions broken down into two or more life cycle stages (e.g., in addition to the total carbon emissions, those arising specifically during the raw material acquisition stage and those during the manufacturing stage were reported separately). The granularity of reported stage level emissions ranges from 2 separate stages per footprint to 9, with an average of 4.1. The value chain breakdown for these footprints was conducted as follows:

*Step 3.a:* Each stage level emission in the raw data was reported with two meta data labels, (i) the associated life cycle stage (e.g., “operation of premises”); and (ii) the “scope”, a nomenclature defined in corporate GHG accounting^32^ (e.g., “scope 1”)^28^. Note that scope 1, 2, 3 is not a nomenclature typically used for LCA-based analyses such as PCF. However, a conceptual mapping of LCA stages such as “transportation” or “manufacturing” to scope 1, 2, and 3 is sometimes introduced^32^, and the Supply Chain Module of CDP’s Climate Change Questionnaire^28^ offers companies to use the scope 1, 2, 3 nomenclature to further characterize each reported life cycle stage. We therefore included the reported scope 1, 2, 3 labels in further identifying the nature of each reported life cycle stage, as follows: The raw data of the 454 footprints included a total of 312 unique combinations of the twin labels of stage and scope. Some were used by many footprints (e.g., “Consumer use | scope 3” was used by 107 footprints) whereas others by very few (e.g., “Other: Packaging/Equipment production | scope 3” was used by only 3 footprints). We created a scheme that mapped each of these 312 twin labels to one of the three value chain stages, upstream, direct operations, and downstream. A more granular (yet consistent across products) breakdown was not possible because of the limited number of stage-level emissions per product provided in the raw data. In addition, each twin-label was specified as “exclusively transportation (yes/no)” and “exclusively end-of-life (yes/no)”. However, for other footprints where stage emissions for transportation [or end-of-life] were not explicitly identified by the company, these emissions are not zero, but rather simply already included as portions of the other reported stages. As an example, a company may report life cycle stage emissions for “distribution and use | scope 3”, meaning this stage includes emissions from downstream transportation along with emissions from other downstream, non-transportation activities. This twin-label was therefore mapped to “downstream”, but not also to “exclusively transportation”. In other words, transportation-related emissions are always included in the upstream and/or downstream portions [and end-of-life emissions in downstream], and sometimes also specified separately. In most cases, the mapping was straight forward based solely on the twin labels reported in the raw data (e.g., “Retail and home storage | scope 3” was mapped to downstream). In some cases, the actual associated product and its other life cycle stages had to be consulted, in order to disambiguate the value chain stage (e.g., “Corrugator | no scope info” was mapped to “direct operations” as it pertained to making a corrugated cardboard box). The full mapping table is provided in *Supplementary Information*.

*Step 3.b*: Of the 454 footprints, 36 included negative stage-level emissions, referring to carbon offsets from recycling^5^. We excluded the emissions for these stages (not however the footprint’s other stage-level data) from the analysis, for the following reasons: (i) They were generally small (~5% or less of the total footprint, i.e., below commonly assumed materiality thresholds for PCFs^33^). (ii) The approach to carbon offsets from recycling is an area of ongoing debate, with offsets contributed sometimes to raw materials (i.e., upstream) and sometimes to end-of-life (i.e., downstream), and subject to strict rules with regards to the quality of the recycled material^5^.

*Step 3.c*: For each of the 454 footprints separately, any stage-level carbon emissions that had been mapped to the same value chain portion in step 3.a (e.g., upstream) were added. This sum was then divided by the sum of all stage-level emissions of that footprint. For each of the 454 footprints, this yielded three percentages, henceforth *%Upstream*, *%Direct Operations*, and *%Downstream* (which by definition add up to 100%). For subsets of the 454 footprints, this also yielded percentages for *Transportation* and/or *End-of-life*.

*Step 3.d:* In the raw data, the total carbon emissions of a particular product were reported in a different section of the questionnaire^28^ than the product’s emissions by life cycle stages. This could lead to discrepancies between the two, but also provided an opportunity to check the accuracy of a product’s reported life cycle stage data by validating it against the product’s reported total emissions: For most products, the sum of separate life cycle stage emissions was between 0.9 and 1.1 of the total footprint. We interpreted this as indicating that the life cycle stage data was complete and reliable. A residual discrepancy of less than 10% would be within the expected error margin of best practice carbon footprints^33–35^, and in many cases was simply due to rounding errors resulting from the limited number of significant digits used by the company when entering raw data into the questionnaire. 10 of the 454 footprints (2%) reported stage level emissions data whose sum was less than 0.9 of the total footprint. This indicates either incomplete life cycle stage data (e.g., the use phase was accidentally omitted when submitting the questionnaire) or simply typos/other errors. Similarly, 23 of the 454 (5%) footprints reported stage level data whose sum was more than 1.1 of the total, indicating typos/other errors in the questionnaire submission. Therefore, for these 33 products, the value chain breakdown was excluded from all further analysis, leaving 421 footprints with value chain breakdowns deemed complete and reliable. Of these 421, 298 further specified the contribution of transportation processes to the total footprint, and 180 further specified the contribution of end-of-life processes.

*Step 3.e:* 104 of the 421 footprints emerged from steps 3.a-d with a value chain breakdown of zero *%Upstream*. This does not mean that these products did not have any upstream-related emissions, but rather that the company’s submission of raw data was not sufficiently granular to separate upstream emissions from direct operations during the above mapping step 3.b. For example, a company may have chosen to report a life cycle stage “Manufacturing | Scope 1, 2, 3” which combines underlying upstream preprocessing by third parties and the reporting company’s own manufacturing into a single life cycle stage. The mapping table in step 3.b slotted such emissions into direct operations, resulting in a bias towards higher emission portions from direct operations. To correct this bias, the value chain breakdown for these 104 footprints was corrected using sector averages from those footprints that did provide more granular stage information in the raw data, as follows: (i) The downstream portion, for each footprint individually, was left as is. (ii) The remainder, i.e., *%Direct Operations* = *1-%Downstream*, was split into *%Upstream* versus *%Direct Operations* according to the average ratio of *%Upstream* versus *%Direct Operations* of all other footprints in the sector that had reported upstream emissions separately. This approach was chosen because it had the following intended effects: (i) The individual *%Downstream* of all 421 footprints remained the same as implied in the raw data; and (ii) the ratios of sector average *%Upstream* versus *%Direct Operations* of the 421 products were the same as for the 317 footprints that included raw data at stage levels sufficiently granular to determine *%Upstream* separate from *%Direct Operations*.

*Treatment of “cradle-to-gate” footprints:* 80 of the 421 footprints were submitted to CDP without any downstream emissions, indicating that they comprise “cradle-to-gate” footprints^5^. As expected, these preferentially occur in those sectors where such life cycle assessments are customary because products are often intermediate business-to-business products rather than finished consumer-facing products. Accordingly, 85% of the 80 footprints are concentrated in only 3 of the 8 sectors: Construction and commercial materials, Packaging for consumer goods, and Chemicals. Unless otherwise indicated, analyses in *Results* include these footprints because: (i) From the perspective of the reporting company, these are complete footprints that include every emission that are under the company’s control; (ii) the larger sample of 866 footprints will likely include other cradle-to-gate footprints beyond the 80 (which could, however, not be identified as such because companies reported them without any stage level data).

### Step 4: Categorizing company-reported reasons for product footprint changes

Of the 866 footprints, a sub-sample of 250 in the CDP raw data includes a percentage that indicates how much a product’s emissions changed vis-à-vis the company’s previous assessment of that product (typically 1–2 years prior). This percentage is included in the questionnaire, irrespective of whether or not the company reported this particular product in the previous year’s response to the questionnaire. For the remaining 616 footprints, this percentage is either left blank (274 footprints) or reported as 0% (342). However, even when reporting 0%, companies typically add a note in the raw data such as “*LCAs do not change from one year to the other if no major changes in processes/manufacturing of the product occur, because LCAs are based on standard databases”*. This means that the actual carbon emissions of the product may very well have changed (e.g., because the energy mix in the manufacturing country changed), but the company simply did not re-assess the product’s carbon emissions. Therefore, for these 342 footprints, the reported 0% was instead interpreted as “*not known”* and therefore not included in the calculated average footprint changes shown in *Results*.

For the 250 footprints, we then grouped the company’s self-reported reason for the footprint change into 1 of 4 categories:

(1) *Carbon emissions changed* (n = 166): This category was assigned to footprints that reportedly changed because changes in the product’s life cycle led to emission changes. Even though there are a large variety of reasons given by companies, such actual change in carbon emissions could always be identified without ambiguity. For example, a company reported a “*change in secondary packaging format from high cone to shrink fill*”.

(2a) *LCA model and/or parameters updated* (n = 25): This category was assigned to footprints that reportedly changed because the LCA model and/or specific parameters in the model were updated (however without any actual change in emissions). Such updates happen frequently, as a consequence of ever improving model and parameter quality in LCA studies^35^, and could be identified in the raw data. For example, a company reported that a “*recalculation has been done with Simapro V8.0 and Ecoinvent V2.2 and V3*”. (2b) *Combination of model/parameters AND carbon emission changes* (n = 21): This category was assigned to footprints that reportedly changed because of a combination of above reasons (1) and (2a). Such cases could be identified in the raw data as well. For example, a company reported that a “*product mix with higher average face weight, an increased use of Nylon 6, and increased energy use were offset by a change in calculation methodology for End of Life impacts resulting in a net decrease in emissions per unit*”. (2c) *Footprint change reported, but without a specified reason* (n = 38): This category was assigned to footprints for which companies had reported the magnitude of the footprint reduction/increase, however without specifying the reason.

Above categories 2a, 2b, and 2c have in common that the footprint change could not be exclusively attributed to actual changes in emissions along the product’s value chain. Therefore, for the analyses in *Results*, categories 2a, 2b, and 2c were combined into a larger sample (n = 84), in order to compare the average magnitude of the change to that of the 166 footprint changes in category 1 which were known to be exclusively due to a change in emissions.

### Statistical analyses and confidence intervals

Averages, standard error of the mean (SEM), correlations, analyses of variance (ANOVA), student t-tests, and linear regressions^36^ were carried out using various standard statistics software. All statistical tests considered the actual size of the respective subsample available for each analysis (Table 1). Value chain breakdown percentages (range: 0–100%) and footprint change percentages (range: −82% (i.e., emission reduction) to +103%) were treated as normal distributions. The distribution of CIs is highly asymmetric, but well approximated by a lognormal distribution of μ = 1.85 and σ = 1.95 (*Supplementary Information)*. Therefore, all statistical analyses of CI were carried out after first transforming to ln(CI). All error bars shown throughout *Results* are SEM. While the upper and lower SEM for lognormally distributed CI are not the same, for simplicity, SEM for CI show the *average* of the upper and lower SEM (which each were determined by assessing the SEM for ln(CI) and converting back to CI). For ANOVA, we report η^2^, the ratio of inter-group variance to total variance of a particular observable.

## Supplementary information

Supplementary Information.

## Data Availability

*PCF-Database* (866 products), which was generated and analyzed during the current study, is available from the corresponding author upon reasonable request.
