# Supplementary material for: Carbon emissions embodied in product value chains and the role of Life Cycle Assessment in curbing them
Source: Sci Rep. 2020 Apr 10;10:6184. doi: 10.1038/s41598-020-62030-x (PMC7148294; doi:10.1038/s41598-020-62030-x)
Supplement: Supplementary file 1 — Supplementary Information. [file 41598_2020_62030_MOESM1_ESM.pdf]

Sources of embodied carbon emissions along product value chains

Christoph J Meinrenken[a*], Daniel Chen[b], Ricardo A. Esparza[a],
Venkat Iyer[a], Sally Paridis[b], Aruna Prasad[c], and Erika Whillas[b]

** Corresponding author (email: cmeinrenken@ei.columbia.edu)*
[a] Columbia University, New York, NY, USA
[b] CoClear, Purchase, NY, USA
[c] University of Pennsylvania, Philadelphia, PA, USA

SUPPLEMENTARY INFORMATION

ABSTRACT

Life cycle-based analyses are considered crucial for designing product value chains towards lower carbon emissions. We have used data reported by companies to CDP for public disclosure to build a database of 866 product carbon footprints (PCFs), from 145 companies, 30 industries, and 28 countries. We used this database to elucidate the breakdown of emissions across products' value chains, how this breakdown varies by industry, and whether the reported emission reductions vary with the granularity of the PCF. For the 866 products, on average 45% of total value chain emissions arise upstream in the supply chain, 23% during the company's direct operations, and 32% downstream. This breakdown varies strongly by industry. Across their lifecycle, the 866 products caused average total emissions of 6 times their own weight, with large variation within and across industries. Reported achievements to reduce emissions varied depending on whether a company had reported a PCF's breakdown to life cycle stages or only the total emissions (10.9% average reduction with breakdown versus 3.7% without). We conclude that a sector-level understanding of emissions, absent of individual PCFs, is insufficient to reliably quantify carbon emissions, and that higher reported emission reductions are accompanied by more granular PCFs.

Correspondence to cmeinrenken@ei.columbia.edu

SuppInfo 1 | CIs

Distribution of Carbon Intensities (CI) well approximated by lognormal distribution

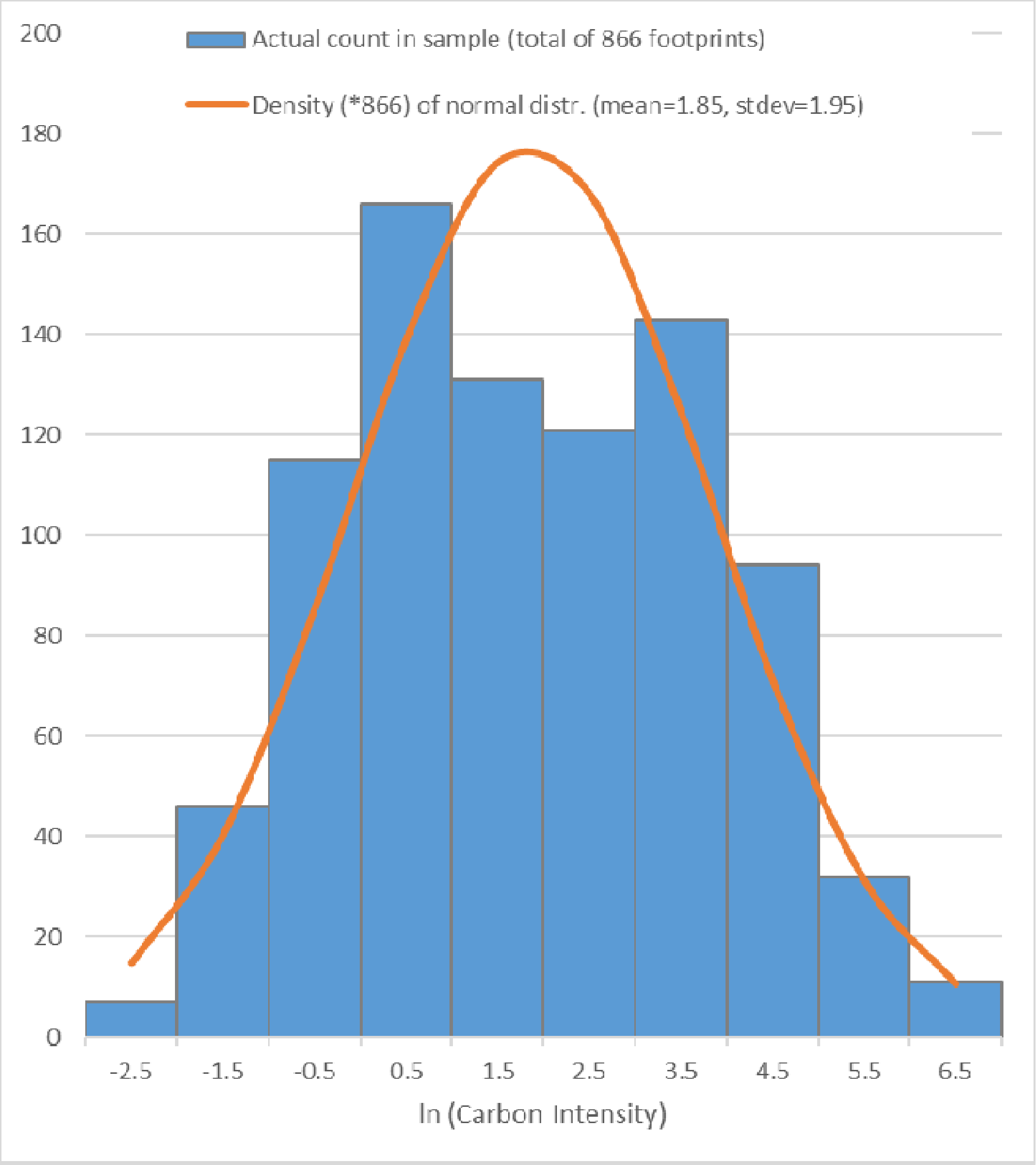

SuppInfo 2 | GICS mapping

CDP GICS Industry Group	CDP GICS Industry	Mapped Sector
Automobiles & Components	Auto Components	Automobiles & components
Automobiles & Components	Automobiles	Automobiles & components
Automobiles & Components	(unknown)*	Automobiles & components
Tires	(unknown)	Automobiles & components
Chemicals	(unknown)	Chemicals
Energy	Oil, Gas & Consumable Fuels	Chemicals
Materials	Chemicals	Chemicals
Capital Goods	Aerospace & Defense	Comm. equipm. & capital goods
Capital Goods	Building Products	Comm. equipm. & capital goods
Capital Goods	Construction & Engineering	Comm. equipm. & capital goods
Capital Goods	Electrical Equipment	Comm. equipm. & capital goods
Capital Goods	Machinery	Comm. equipm. & capital goods
Capital Goods	Trading Companies & Distributors	Comm. equipm. & capital goods
Construction & Engineering	(unknown)	Comm. equipm. & capital goods
Electric Utilities & Independent Power Producers & Energy Traders (including fossil, alternative and nuclear energy)	(unknown)	Comm. equipm. & capital goods
Electrical Equipment and Machinery	(unknown)	Comm. equipm. & capital goods
Gas Utilities	(unknown)	Comm. equipm. & capital goods
Pharmaceuticals, Biotechnology & Life Sciences	Life Sciences Tools & Services	Comm. equipm. & capital goods
Trading Companies & Distributors and Commercial Services & Supplies	(unknown)	Comm. equipm. & capital goods
Utilities	Electric Utilities	Comm. equipm. & capital goods
Utilities	Gas Utilities	Comm. equipm. & capital goods
Utilities	Multi-Utilities	Comm. equipm. & capital goods
Utilities	Water Utilities	Comm. equipm. & capital goods
Media	Media	Computer, IT & telecom
Media	(unknown)	Computer, IT & telecom
Semiconductors & Semiconductor Equipment	Semiconductors & Semiconductor Equipment	Computer, IT & telecom
Semiconductors & Semiconductors Equipment	(unknown)	Computer, IT & telecom
Software & Services	IT Services	Computer, IT & telecom
Software & Services	(unknown)	Computer, IT & telecom
Software & Services	Software	Computer, IT & telecom
Technology Hardware & Equipment	Communications Equipment	Computer, IT & telecom
Technology Hardware & Equipment	Computers & Peripherals	Computer, IT & telecom
Technology Hardware & Equipment	Electronic Equipment, Instruments & Components	Computer, IT & telecom
Technology Hardware & Equipment	(unknown)	Computer, IT & telecom
Technology Hardware & Equipment	Office Electronics	Computer, IT & telecom
Telecommunication Services	Diversified Telecommunication Services	Computer, IT & telecom
Telecommunication Services	(unknown)	Computer, IT & telecom
Telecommunication Services	Wireless Telecommunication Services	Computer, IT & telecom
Forest and Paper Products - Forestry, Timber, Pulp and Paper, Rubber	(unknown)	Construction & commercial materials
Materials	Construction Materials	Construction & commercial materials
Materials	Metals & Mining	Construction & commercial materials
Materials	Paper & Forest Products	Construction & commercial materials
Mining - Iron, Aluminum, Other Metals	(unknown)	Construction & commercial materials
Food & Beverage Processing	(unknown)	Food & Beverage
Food & Staples Retailing	Food & Staples Retailing	Food & Beverage
Food & Staples Retailing	(unknown)	Food & Beverage
Food, Beverage & Tobacco	Beverages	Food & Beverage
Food, Beverage & Tobacco	Food Products	Food & Beverage
Food, Beverage & Tobacco	Tobacco	Food & Beverage
Tobacco	(unknown)	Food & Beverage
Building Products	(unknown)	Home durables, textiles, & equipment
Commercial & Professional Services	Commercial Services & Supplies	Home durables, textiles, & equipment
Consumer Durables & Apparel	Household Durables	Home durables, textiles, & equipment
Consumer Durables & Apparel	Leisure Equipment & Products	Home durables, textiles, & equipment
Consumer Durables & Apparel	Textiles, Apparel & Luxury Goods	Home durables, textiles, & equipment
Consumer Durables, Household and Personal Products	(unknown)	Home durables, textiles, & equipment
Retailing	(unknown)	Home durables, textiles, & equipment
Retailing	Specialty Retail	Home durables, textiles, & equipment
Textiles, Apparel, Footwear and Luxury Goods	(unknown)	Home durables, textiles, & equipment
Containers & Packaging	(unknown)	Packaging for consumer goods
Household & Personal Products	Personal Products	Packaging for consumer goods
Materials	Containers & Packaging	Packaging for consumer goods

* *The field "GICS Industry" was added to the CDP Questionnaire only for years 2014 onwards.*

SuppInfo 3 | ValueChain mapping

CDP SM32b LCA Stage	CDP SM32b Scope	Value Chain Breakdown	Exclusively Transport	Exclusively End-of-life
Assembly	(unknown) *	Direct Operations	No	No
Assembly	Scope 1	Direct Operations	No	No
Assembly	Scope 1 & 2	Direct Operations	No	No
Assembly	Scope 1, 2, & 3	Direct Operations	No	No
Assembly	Scope 2	Direct Operations	No	No
Assembly	Scope 3	Direct Operations	No	No
Assembly and finishing of product	(unknown)	Direct Operations	No	No
Bagging	(unknown)	Direct Operations	No	No
Beer Production and processing	(unknown)	Direct Operations	No	No
BLANK	BLANK	Direct Operations	No	No
BLANK	Scope 1	Direct Operations	No	No
BLANK	Scope 2	Direct Operations	No	No
BLANK	Scope 3	Direct Operations	No	No
Boiler, coffee maker and cup manufacturing: For more specific details please see Humbert et al. (2009) Life cycle assessment of spray dried soluble coffee and comparison with alternatives (drip filter and capsule espresso)	(unknown)	Downstream	No	No
Box production	(unknown)	Direct Operations	No	No
Business Travel	(unknown)	Direct Operations	No	No
Casting	(unknown)	Direct Operations	No	No
Consumer use	BLANK	Downstream	No	No
Consumer use	(unknown)	Downstream	No	No
Consumer use	Scope 1 & 2	Downstream	No	No
Consumer use	Scope 1, 2, & 3	Downstream	No	No
Consumer use	Scope 3	Downstream	No	No
Converting	(unknown)	Direct Operations	No	No
Converting (including production of raw materials used, fuels consumption, electricity consumption, transport of raw materials)	(unknown)	Direct Operations	No	No
Corrugator	(unknown)	Direct Operations	No	No
Cradle to gate	(unknown)	Direct Operations	No	No
Cradle to gate	Other: Cradle to gate	Direct Operations	No	No
Cradle to gate	Scope 1	Direct Operations	No	No
Cradle to gate	Scope 1 & 2	Direct Operations	No	No
Cradle to gate	Scope 1, 2, & 3	Direct Operations	No	No
Cradle to gate	Scope 2	Direct Operations	No	No
Cradle to gate	Scope 3	Upstream	No	No
Cradle to grave	Scope 1, 2, & 3	Downstream	No	No
Cradle-to-Gate	(unknown)	Direct Operations	No	No
Cultivation: For more specific details please see Humbert et al. (2009) Life cycle assessment of spray dried soluble coffee and comparison with alternatives (drip filter and capsule espresso)	(unknown)	Upstream	No	No
Customer Use (5 years)	(unknown)	Downstream	No	No
Delivery and Installation	(unknown)	Downstream	No	No
Delivery of components	(unknown)	Downstream	Yes	No
Delivery: For more specific details please see Humbert et al. (2009) Life cycle assessment of spray dried soluble coffee and comparison with alternatives (drip filter and capsule espresso)	(unknown)	Downstream	Yes	No
Disposal	(unknown)	Downstream	No	Yes
Disposal and Recycling	(unknown)	Downstream	No	Yes
Dispose	(unknown)	Downstream	No	Yes
Distribution	(unknown)	Downstream	Yes	No
Distribution	Scope 1	Downstream	Yes	No
Distribution	Scope 1, 2, & 3	Downstream	Yes	No
Distribution	Scope 3	Downstream	Yes	No
Distribution (Product)	(unknown)	Downstream	Yes	No
Distribution stage	(unknown)	Downstream	Yes	No
Distribution/Warehousing	(unknown)	Downstream	Yes	No

Distribution: For more specific details please see Humbert et al. (2009) Life cycle assessment of spray dried soluble coffee and comparison with alternatives (drip filter and capsule espresso)	(unknown)	Downstream	Yes	No
Downstream Transport	(unknown)	Downstream	Yes	No
Downstream Transportation & Distribution	(unknown)	Downstream	Yes	No
Electric Motor manufacturing	(unknown)	Direct Operations	No	No
Electric Motor useful life (from operation until the end of the motor life). Considering the 10-year life cycle of the electric motor	(unknown)	Downstream	No	No
Emissions in use	(unknown)	Downstream	No	No
End of Life	(unknown)	Downstream	No	Yes
End of Life (Disp & Recycling)	(unknown)	Downstream	No	Yes
End of life (in kg CO2-e / kg product)	(unknown)	Downstream	No	Yes
End Of Life (Minus number)	(unknown)	Downstream	No	Yes
End of life (recycling, cut-off)	(unknown)	Downstream	No	Yes
End of life/Final disposal	BLANK	Downstream	No	Yes
End of life/Final disposal	Scope 1 & 2	Downstream	No	Yes
End of life/Final disposal	Scope 1, 2, & 3	Downstream	No	Yes
End of life/Final disposal	Scope 3	Downstream	No	Yes
End-of-Life	(unknown)	Downstream	No	Yes
End-of-life (negative emissions)	(unknown)	Downstream	No	Yes
End-of-Life (This phase actually had a CO2 impact of -4 due to recycling, so we deducted that impact from the production phase to the totals would match)	(unknown)	Downstream	No	Yes
Energy/Fuel	BLANK	Direct Operations	No	No
Energy/Fuel	Scope 1	Direct Operations	No	No
Energy/Fuel	Scope 1 & 2	Direct Operations	No	No
Energy/Fuel	Scope 2	Direct Operations	No	No
Energy/Fuel	Scope 3	Direct Operations	No	No
Feedsotck	(unknown)	Upstream	No	No
Feedstock	(unknown)	Upstream	No	No
fishing - longline albacore	(unknown)	Upstream	No	No
fishing - purse seine	(unknown)	Upstream	No	No
Industrial process - raw materials to final product storage	(unknown)	Direct Operations	No	No
Ingredients	(unknown)	Upstream	No	No
Ingredients (in kg CO2-e / kg product)	(unknown)	Upstream	No	No
Input Materials and Supplies	(unknown)	Upstream	No	No
Installation	(unknown)	Downstream	No	No
Logistics	(unknown)	Downstream	Yes	No
Logistics (in kg CO2e / kg product)	(unknown)	Downstream	Yes	No
Logistics (in kg CO2-e / kg product)	(unknown)	Downstream	Yes	No
Making components	(unknown)	Direct Operations	No	No
Making raw materials	(unknown)	Upstream	No	No
Manufacture	(unknown)	Direct Operations	No	No
Manufacture of AVK finished components	(unknown)	Direct Operations	No	No
Manufacture of materials	(unknown)	Upstream	No	No
Manufacture of product	(unknown)	Direct Operations	No	No
Manufacture of supplier components	(unknown)	Upstream	No	No
Manufacturing	BLANK	Direct Operations	No	No
Manufacturing	(unknown)	Direct Operations	No	No
Manufacturing	Scope 1	Direct Operations	No	No
Manufacturing	Scope 1 & 2	Direct Operations	No	No
Manufacturing	Scope 1, 2, & 3	Direct Operations	No	No
Manufacturing	Scope 2	Direct Operations	No	No
Manufacturing	Scope 3	Direct Operations	No	No
Manufacturing (including raw material extraction and Interface manufacturing process)	(unknown)	Direct Operations	No	No
Manufacturing and Operations	(unknown)	Direct Operations	No	No
Manufacturing of CRU	(unknown)	Direct Operations	No	No
Manufacturing of Printer	(unknown)	Direct Operations	No	No
Manufacturing process	(unknown)	Direct Operations	No	No
Manufacturing stage	(unknown)	Direct Operations	No	No
Material	(unknown)	Upstream	No	No
Material (steel, cast iron, zinc)	(unknown)	Upstream	No	No
Material acquisition	Scope 1	Upstream	No	No

Material acquisition	Scope 1 & 2	Upstream	No	No
Material acquisition	Scope 1, 2, & 3	Upstream	No	No
Material acquisition	Scope 3	Upstream	No	No
Material/Recycle	(unknown)	Downstream	No	Yes
Materials and Manufacturing	(unknown)	Direct Operations	No	No
Materials input in LCD and LCM process	(unknown)	Upstream	No	No
Materials stage	(unknown)	Upstream	No	No
Milk (in kg CO2e / kg product)	(unknown)	Upstream	No	No
Nokia factory	(unknown)	Direct Operations	No	No
Operation of premises	Scope 1 & 2	Direct Operations	No	No
Operation of premises	Scope 2	Downstream	No	No
Operation of premises	Scope 3	Downstream	No	No
Other raw materials (in kg CO2e / kg product)	(unknown)	Upstream	No	No
Other:	Scope 1, 2, & 3	Direct Operations	No	No
Other:	Scope 3	Downstream	No	No
Other: Agriculture	Scope 3	Upstream	No	No
Other: Beverage Production	Scope 1, 2, & 3	Direct Operations	No	No
Other: Beverage Production	Scope 3	Direct Operations	No	No
Other: Combustion	(unknown)	Downstream	No	No
Other: Combustion	Scope 1	Downstream	No	No
Other: Combustion	Scope 3	Downstream	No	No
Other: Consumable Use (toner, and print cartridge – excludes paper)	Scope 1, 2, & 3	Downstream	No	No
Other: Consumer Use & Maintenance	Scope 1, 2, & 3	Downstream	No	No
Other: Consumer use (3 years)	BLANK	Downstream	No	No
Other: Consumer use (three years)	BLANK	Downstream	No	No
Other: Delivery & Installation	Scope 3	Downstream	No	No
Other: Distribution and Marketplace	Scope 3	Downstream	Yes	No
Other: Electricity purchased for remote properties (Distribution centres, offices etc.)	Scope 2	Direct Operations	No	No
Other: End of Life	Scope 3	Downstream	No	Yes
Other: End of life and recycling	Scope 3	Downstream	No	Yes
Other: End of Life Energy Recovery	Scope 3	Downstream	No	Yes
Other: EOL	Scope 2	Downstream	No	Yes
Other: Extraction and Liquefaction	Scope 3	Upstream	No	No
Other: Facility	(unknown)	Direct Operations	No	No
Other: Facility	Scope 1	Direct Operations	No	No
Other: Facility	Scope 3	Direct Operations	No	No
Other: Fuel used in remote properties (Distribution centres, offices etc.)	Scope 1	Downstream	No	No
Other: Handling	Scope 3	Downstream	No	No
Other: Installation	Scope 1, 2, & 3	Downstream	No	No
Other: Installation	Scope 3	Downstream	No	No
Other: Losses of CO2 (product) during manufacturing	Scope 1	Direct Operations	No	No
Other: Losses of CO2 used for carbonation of the beverages during manufacturing.	Scope 1	Direct Operations	No	No
Other: Maintenance	Scope 3	Downstream	No	No
Other: Manufacturing & Packaging	Scope 3	Upstream	No	No
Other: Manufacturing of Customer Replacement Unit	Scope 1 & 2	Direct Operations	No	No
Other: Manufacturing of Printer	Scope 1 & 2	Direct Operations	No	No
Other: Manufacturing, Packaging	Scope 3	Upstream	No	No
Other: Milling	Scope 1, 2, & 3	Direct Operations	No	No
Other: milling process	Scope 1, 2, & 3	Direct Operations	No	No
Other: Packaging / Equipment Production	Scope 3	Upstream	No	No
Other: primary packaging end of life	Scope 1, 2, & 3	Downstream	No	Yes
Other: Production of materials composing the product	Scope 3	Upstream	No	No
Other: Purchased goods and services	Scope 3	Upstream	No	No
Other: Raw Material	Scope 3	Upstream	No	No
Other: Raw material production	Scope 1, 2, & 3	Upstream	No	No
Other: Raw Materials (Agriculture and processing)	Scope 1, 2, & 3	Upstream	No	No
Other: Raw materials production	Scope 1, 2, & 3	Upstream	No	No
Other: Refurbishment	Scope 1, 2, & 3	Direct Operations	No	No
Other: Repair	Scope 3	Downstream	No	No
Other: Retail and Home storage	Scope 3	Downstream	No	No

Other: Storage, transportation	Scope 3	Downstream	Yes	No
Other: storage, transportation, manufacturing, packaging	Scope 3	Upstream	No	No
Other: Transport	Scope 3	Downstream	Yes	No
Other: Transportation & Storage	Scope 3	Downstream	Yes	No
Other: upstream to Tetra Pak operations	Scope 3	Upstream	No	No
Other: urchased goods and services	Scope 3	Upstream	No	No
Other: Use	Scope 2	Downstream	No	No
Other: Use - Washing & Refrigeration	Scope 3	Downstream	No	No
Other: Use & Maintenance	Scope 1, 2, & 3	Downstream	No	No
Other: Use electricity	Scope 3	Downstream	No	No
Other: Use of sold products, coal combustion	Scope 3	Downstream	No	No
Overheads: For more specific details please see Humbert et al. (2009) Life cycle assessment of spray dried soluble coffee and comparison with alternatives (drip filter and capsule espresso)	(unknown)	Direct Operations	No	No
Packaging	(unknown)	Upstream	No	No
Packaging	Scope 1 & 2	Direct Operations	No	No
Packaging	Scope 1, 2, & 3	Upstream	No	No
Packaging	Scope 3	Upstream	No	No
Packaging (in kg CO2e / kg product)	(unknown)	Upstream	No	No
Packaging (in kg CO2-e / kg product)	(unknown)	Upstream	No	No
Packaging (material extraction and processing)	(unknown)	Upstream	No	No
Packaging and product end of life (in kg CO2e / kg product)	(unknown)	Downstream	No	Yes
Packaging: For more specific details please see Humbert et al. (2009) Life cycle assessment of spray dried soluble coffee and comparison with alternatives (drip filter and capsule espresso)	(unknown)	Upstream	No	No
Paper mill	(unknown)	Upstream	No	No
Pipe extrusion	(unknown)	Direct Operations	No	No
Pipe transport	(unknown)	Downstream	Yes	No
Polymerization	(unknown)	Direct Operations	No	No
Pre-manufacturing	(unknown)	Upstream	No	No
Pre-processing	Scope 1 & 2	Direct Operations	No	No
Processing	BLANK	Direct Operations	No	No
Processing	Scope 1	Direct Operations	No	No
Processing	Scope 1 & 2	Direct Operations	No	No
Processing	Scope 1, 2, & 3	Direct Operations	No	No
Processing	Scope 3	Downstream	No	No
processing - loining and canning	(unknown)	Direct Operations	No	No
Processing: For more specific details please see Humbert et al. (2009) Life cycle assessment of spray dried soluble coffee and comparison with alternatives (drip filter and capsule espresso)	(unknown)	Direct Operations	No	No
producing process	(unknown)	Direct Operations	No	No
Product Production (Product)	(unknown)	Direct Operations	No	No
Product usage	(unknown)	Downstream	No	No
Product Use	(unknown)	Downstream	No	No
Production	BLANK	Direct Operations	No	No
Production	(unknown)	Direct Operations	No	No
Production	Scope 1	Direct Operations	No	No
Production	Scope 1 & 2	Direct Operations	No	No
Production	Scope 1, 2, & 3	Direct Operations	No	No
Production	Scope 2	Direct Operations	No	No
Production	Scope 3	Upstream	No	No
Production (Actual impact: 58 kg CO2, combined with the -4 impact of End-of-Life)	(unknown)	Direct Operations	No	No
Production (in kg CO2e / kg product)	(unknown)	Direct Operations	No	No
Production (in kg CO2-e / kg product)	(unknown)	Direct Operations	No	No
Production (material, manufacturing)	(unknown)	Direct Operations	No	No
Production of papers (including production of raw materials used, fuels consumption, electricity consumption, transport of raw materials)	(unknown)	Direct Operations	No	No
Products manufacturing	(unknown)	Direct Operations	No	No
raw materail to final product at the gate	(unknown)	Direct Operations	No	No
Raw Material Acquisition/Reprocessing	(unknown)	Upstream	No	No

Raw material and parts manufacturing	(unknown)	Upstream	No	No
Raw material for package and opening	(unknown)	Upstream	No	No
Raw material Production (Product)	(unknown)	Upstream	No	No
Raw material production stage	(unknown)	Upstream	No	No
Raw material Production(Product)	(unknown)	Upstream	No	No
Raw Materials	(unknown)	Upstream	No	No
Raw Materials (Agriculture and processing)	(unknown)	Upstream	No	No
raw materials and acquisition	(unknown)	Upstream	No	No
Recycling	(unknown)	Downstream	No	Yes
Recycling	Scope 1, 2, & 3	Downstream	No	Yes
Recycling	Scope 3	Downstream	No	Yes
Refrigeration	(unknown)	Downstream	No	No
Refurbishment	(unknown)	Direct Operations	No	No
Repair	(unknown)	Downstream	No	No
Resin production	(unknown)	Upstream	No	No
Resin transport	(unknown)	Upstream	Yes	No
Retail / home use (in kg CO2e / kg product)	(unknown)	Downstream	No	No
Retail / home use (in kg CO2-e / kg product)	(unknown)	Downstream	No	No
Scope 1	(unknown)	Direct Operations	No	No
Scope 1: Direct GHG emissions from sources that are owned/controlled by us	(unknown)	Direct Operations	No	No
Scope 2	(unknown)	Direct Operations	No	No
Scope 2: GHG emissions from the generation of purchased electricity.	(unknown)	Direct Operations	No	No
Scope 3 emissions from pur´hased goods and services	(unknown)	Upstream	No	No
Scope 3 emissions from purchased goods & services	(unknown)	Upstream	No	No
Scope 3 emissions from purchased goods and services	(unknown)	Upstream	No	No
Scope 3 emissions from upstream transportation & distribution	(unknown)	Upstream	Yes	No
Scope 3 upstream	(unknown)	Upstream	No	No
Scope 3: Purchased materials	(unknown)	Upstream	No	No
Scope 3: Transportation	(unknown)	Downstream	Yes	No
Sourcing & Extract	(unknown)	Upstream	No	No
Storage	Scope 1	Downstream	No	No
Storage	Scope 1, 2, & 3	Downstream	No	No
Storage	Scope 3	Downstream	No	No
Sugar beet supply - field preparation to factory gate	(unknown)	Upstream	No	No
Transport	(unknown)	Downstream	Yes	No
Transport components	(unknown)	Downstream	Yes	No
Transport Feedstock	(unknown)	Upstream	Yes	No
Transport of raw materials	(unknown)	Upstream	Yes	No
transportaiton (shipping and trucking)	(unknown)	Downstream	Yes	No
Transportation	BLANK	Downstream	Yes	No
Transportation	(unknown)	Downstream	Yes	No
Transportation	Other: Scope 1 & 3	Downstream	Yes	No
Transportation	Scope 1	Downstream	Yes	No
Transportation	Scope 1, 2, & 3	Downstream	Yes	No
Transportation	Scope 2	Downstream	Yes	No
Transportation	Scope 3	Downstream	Yes	No
transportation (shipping and trucking)	(unknown)	Downstream	Yes	No
Treatment: For more specific details please see Humbert et al. (2009) Life cycle assessment of spray dried soluble coffee and comparison with alternatives (drip filter and capsule espresso)	(unknown)	Direct Operations	No	No
Upstream Transport	(unknown)	Upstream	Yes	No
Upstream Transportation & Distribution	(unknown)	Upstream	Yes	No
Usage (5 years)	(unknown)	Downstream	No	No
Usage stage	(unknown)	Downstream	No	No
USB & Packaging Assembly	(unknown)	Direct Operations	No	No
USB & Packaging Raw Materials & Manufacture	(unknown)	Upstream	No	No
Use	(unknown)	Downstream	No	No
Use (3 years)	(unknown)	Downstream	No	No
Use (3-year useful life)	(unknown)	Downstream	No	No
Use (Electricity)	(unknown)	Downstream	No	No

Use and End-of-Life : For more specific details please see Humbert et al. (2009) Life cycle assessment of spray dried soluble coffee and comparison with alternatives (drip filter and capsule espresso) Note: End-of-Life has been aggregated to Use since th	(unknown)	Downstream	No	No
Use at Customer Site	(unknown)	Downstream	No	No
Use Electricity	(unknown)	Downstream	No	No
use phase	(unknown)	Downstream	No	No
Use Phase (lifetime 15 years, Energy consumption 251 KWh/year)	(unknown)	Downstream	No	No
Use phase and end of life phase	(unknown)	Downstream	No	No
Use(Consumption Life Cycle	(unknown)	Downstream	No	No
Use(Consumption Life Cycle)	(unknown)	Downstream	No	No
Use(Electricity)	(unknown)	Downstream	No	No
Utilization	(unknown)	Downstream	No	No
Waste	Scope 1 & 2	Downstream	No	Yes
Waste	Scope 3	Downstream	No	Yes
Waste/recycling stage	(unknown)	Downstream	No	Yes
1. Beverage Production: Pumping and treatment of the drinking water either at a municipal water plant or at a bottled beverage production facility. Transportation of all water production materials up to the point of manufacture is included in this phase.	(unknown)	Direct Operations	No	No
1.Major component suppliers stage:6.84	(unknown)	Upstream	No	No
1a.Packaging production for France. For more specific details please see Humbert et al. (2009) Life cycle assessment of two baby food packaging alternatives: glass jars vs. plastic pots.	(unknown)	Upstream	No	No
1b.Distribution for France. For more specific details please see Humbert et al. (2009) Life cycle assessment of two baby food packaging alternatives: glass jars vs. plastic pots.	(unknown)	Downstream	Yes	No
1c.Product assembly and preservation process for France. For more specific details please see Humbert et al. (2009) Life cycle assessment of two baby food packaging alternatives: glass jars vs. plastic pots.	(unknown)	Direct Operations	No	No
1d.End of life of packaging for France. For more specific details please see Humbert et al. (2009) Life cycle assessment of two baby food packaging alternatives: glass jars vs. plastic pots.	(unknown)	Downstream	No	Yes
2. Packaging / Equipment Production: Extraction of raw materials and production of the drinking container and drinking glasses used. Transportation of all production materials for these components up to the point of manufacture are included in this phase	(unknown)	Upstream	No	No
2.sales transport stage:1.32	(unknown)	Downstream	Yes	No
2a.Packaging production for Spain. For more specific details please see Humbert et al. (2009) Life cycle assessment of two baby food packaging alternatives: glass jars vs. plastic pots.	(unknown)	Upstream	No	No
2b. Distribution for Spain. For more specific details please see Humbert et al. (2009) Life cycle assessment of two baby food packaging alternatives: glass jars vs. plastic pots.	(unknown)	Downstream	Yes	No
2c. Product assembly and preservation process for Spain. For more specific details please see Humbert et al. (2009) Life cycle assessment of two baby food packaging alternatives: glass jars vs. plastic pots.	(unknown)	Direct Operations	No	No
2d.End of life of packaging for Spain. For more specific details please see Humbert et al. (2009) Life cycle assessment of two baby food packaging alternatives: glass jars vs. plastic pots.	(unknown)	Downstream	No	Yes

3. Use - Transport: All transportation occurring in the system beginning with the immediate suppliers to the manufacturers of beverage products and drinking containers, and ending with the transport of all materials to end-of-life. Also included are tran	(unknown)	Downstream	Yes	No
3.LCD Array Cell manufacturing stage:2.88	(unknown)	Direct Operations	No	No
3a. Packaging production for Germany. For more specific details please see Humbert et al. (2009) Life cycle assessment of two baby food packaging alternatives: glass jars vs. plastic pots.	(unknown)	Upstream	No	No
3b.Distribution for Germany. For more specific details please see Humbert et al. (2009) Life cycle assessment of two baby food packaging alternatives: glass jars vs. plastic pots.	(unknown)	Downstream	Yes	No
3c. Product assembly and preservation process for Germany. For more specific details please see Humbert et al. (2009) Life cycle assessment of two baby food packaging alternatives: glass jars vs. plastic pots.	(unknown)	Direct Operations	No	No
3d. End of life of packaging for Germany. For more specific details please see Humbert et al. (2009) Life cycle assessment of two baby food packaging alternatives: glass jars vs. plastic pots.	(unknown)	Downstream	No	Yes
4. Distribution and Marketplace: Activities related to marketing the products, including operation of retail outlets and/or coolers.	(unknown)	Downstream	Yes	No
4.LCD Module assembly stage:0.96	(unknown)	Direct Operations	No	No
6. Use and End-of-Life : as the system doesn't allow for negative figures, we have aggregated Use phase and End-of-life phase. Use: Washing & Refrigeration Refrigeration of the water at the user's home. Commercial coolers are included in the Distributio	(unknown)	Downstream	No	No

** Not all fields were included in CDP questionnaire in all years.*
